# Supplementary material for: Resilience for Undergraduate Students: Development and Evaluation of a Theory-Driven, Evidence-Based and Learner Centered Digital Resilience Skills Enhancement (RISE) Program
Source: Int J Environ Res Public Health. 2022 Oct 5;19(19):12729. doi: 10.3390/ijerph191912729 (PMC9564580; doi:10.3390/ijerph191912729)
Supplement: Supplementary file 1 [file ijerph-19-12729-s001.zip › ijerph-1943049 - supplementary.pdf]

Table S1. Results of content validation of RISE program among five experts.

Items		Expert 1	Expert 2	Expert 3	Expert 4	Expert 5	Mean	Percentage
Indicate the number which you feel best represents the site: 1 = disagree, 2 = agree, or not applicable (N/A). Add up the total points scored for each page at the bottom of each page.								
**Content**								
	1. The purpose of the site is clearly stated or may be clearly inferred.	2/2	2/2	2/2	2/2	2/2	2	100%
	2. The information covered does not appear to be an "infomercial"	2/2	2/2	2/2	2/2	2/2	2	100%
	3. There is no bias evident.	2/2	2/2	2/2	2/2	2/2	2	100%
	4. If the site is opinionated, the author discusses all sides of the issue, giving each due respect.	2/2	2/2	2/2	2/2	2/2	2	100%
	5. All aspects of the subject are covered adequately.	2/2	2/2	2/2	2/2	2/2	2	100%
	6. External Links are provided to fully cover the subject	2/2	2/2	2/2	2/2	2/2	2	100%
**Accuracy**								
	1. The information is accurate	2/2	NA	2/2	2/2	2/2	2	100%
	2. Sources are clearly documented.	2/2	NA	2/2	2/2	2/2	2	100%
	3. The web site states that it subscribes to HON code principles	1/2	1/2	1/2	1/2	NA	1	50%
**Author**								
	1. The site is sponsored by or is associated with an institution or organization.	2/2	2/2	2/2	2/2	2/2	2	100%
	2. For sites created by an individual, author's/editor's credentials are clearly stated.	2/2	2/2	2/2	2/2	2/2	2	100%
	3. Contact information for the author/editor or webmaster is included	2/2	2/2	2/2	2/2	2/2	2	100%
**Currency**								
	1. The date of publication is clearly posted.	2/2	2/2	2/2	2/2	2/2	2	100%
	2. The revision date is recent enough to account for changes in the field.	2/2	1/2	2/2	2/2	2/2	1.8	90%
**Audience**								

	1. The type of audience the author is addressing is evident	2/2	2/2	2/2	2/2	2/2	2	100%
	2. The level of detail is appropriate for the audience.	2/2	2/2	2/2	2/2	2/2	2	100%
	3. The reading level is appropriate for the audience.	2/2	2/2	2/2	2/2	2/2	2	100%
	4. Technical terms are appropriate for the audience.	2/2	2/2	2/2	2/2	2/2	2	100%
**Navigation**								
	1. Internal links add to the usefulness of the site.	2/2	NA	2/2	2/2	NA	2	100%
	2. Information can be retrieved in a timely manner.	2/2	NA	2/2	2/2	NA	2	100%
	3. A search mechanism is necessary to make this site useful.	2/2	NA	2/2	2/2	NA	2	100%
	4. A search mechanism is provided.	2/2	NA	2/2	2/2	NA	2	100%
	5. The site is organized in a logical manner, facilitating the location of information.	2/2	NA	2/2	2/2	NA	2	100%
	6. Any software necessary to use the page has links to download software from the Internet.	2/2	NA	2/2	2/2	NA	2	100%
**Links**								
	1. Links are relevant and appropriate for this site.	2/2	2/2	2/2	2/2	NA	2	100%
	2. Links are operable.	2/2	2/2	2/2	2/2	NA	2	100%
	3. Links are current enough to account for changes in the field.	2/2	2/2	2/2	2/2	NA	2	100%
	4. Links are appropriate for the audience	2/2	2/2	2/2	2/2	NA	2	100%
	5. Links connect to reliable information from reliable sources.	2/2	2/2	2/2	2/2	NA	2	100%
	6. Links are provided to organizations that should be represented.	2/2	2/2	2/2	2/2	NA	2	100%
	7. Links are relevant and appropriate for this site.	2/2	2/2	2/2	2/2	NA	2	100%
**Structure**								
	1. Educational graphics and art add to the usefulness of the site.	2/2	2/2	2/2	2/2	NA	2	100%
	2. Decorative graphics do not significantly slow down-loading	2/2	2/2	2/2	2/2	NA	2	100%
	3. Text-only option is available for text-only Web browsers.	2/2	2/2	2/2	2/2	NA	2	100%
	4. Usefulness of site does not suffer when using text-only option.	2/2	2/2	2/2	2/2	NA	2	100%
	5. Options are available for disabled persons	2/2	2/2	2/2	2/2	NA	2	100%
	6. If audio and video are components of the site, and cannot be accessed, the information on the site is still complete.	2/2	2/2	2/2	2/2	NA	2	100%

	Total score	71/72=98.6%	62/64=96.9%	71/72=98.6%	71/72=98.6%	34/34=100%	1.98	99.2%
	Rating	Excellent	Excellent	Excellent	Excellent	Excellent		

Table S2. Open-ended questions.

**Domains**	**Questions**
Acceptability and satisfaction	• What is your experience of using RISE?
	• Did you experience any issues while using RISE?
	• What are your thoughts about the duration of the RISE program?
	• What are your thoughts about the take home tasks?
Appropriateness	• Were there any sessions that were useful?
	• Were there any sessions that were not useful?
	• Are there any other strategies that we could include in future RISE programs?
Demand and actual use	• Did the training influence your resilience?
	• Did the training influence your social support?
	• Did the training influence the way you learn?
	• Are there any suggestions to improve the training program?
Probing questions	• Can you elaborate on why you have stated that response?
	• Why did you say that?

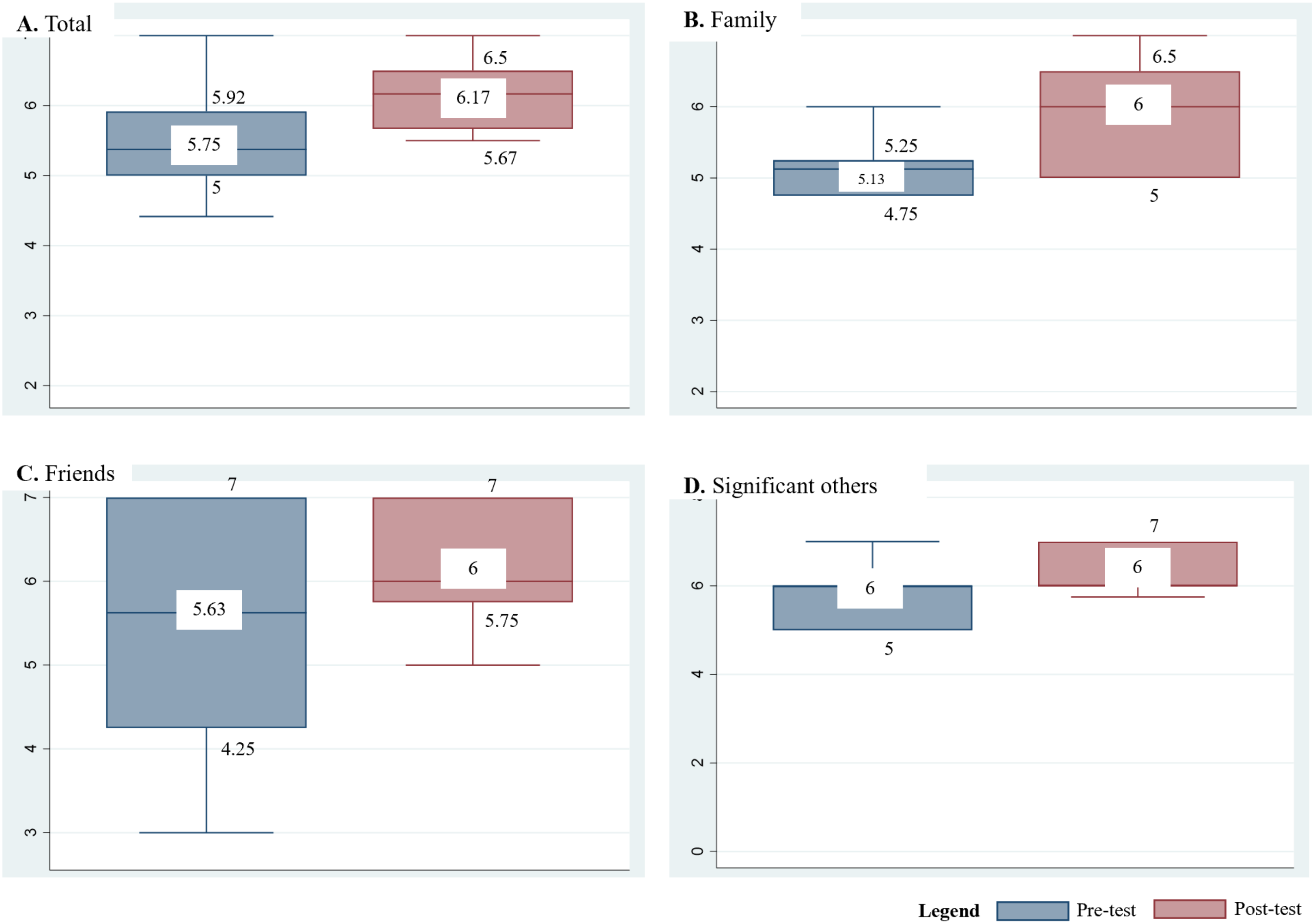

Figure S1. Box plots of social support and subscale scores.

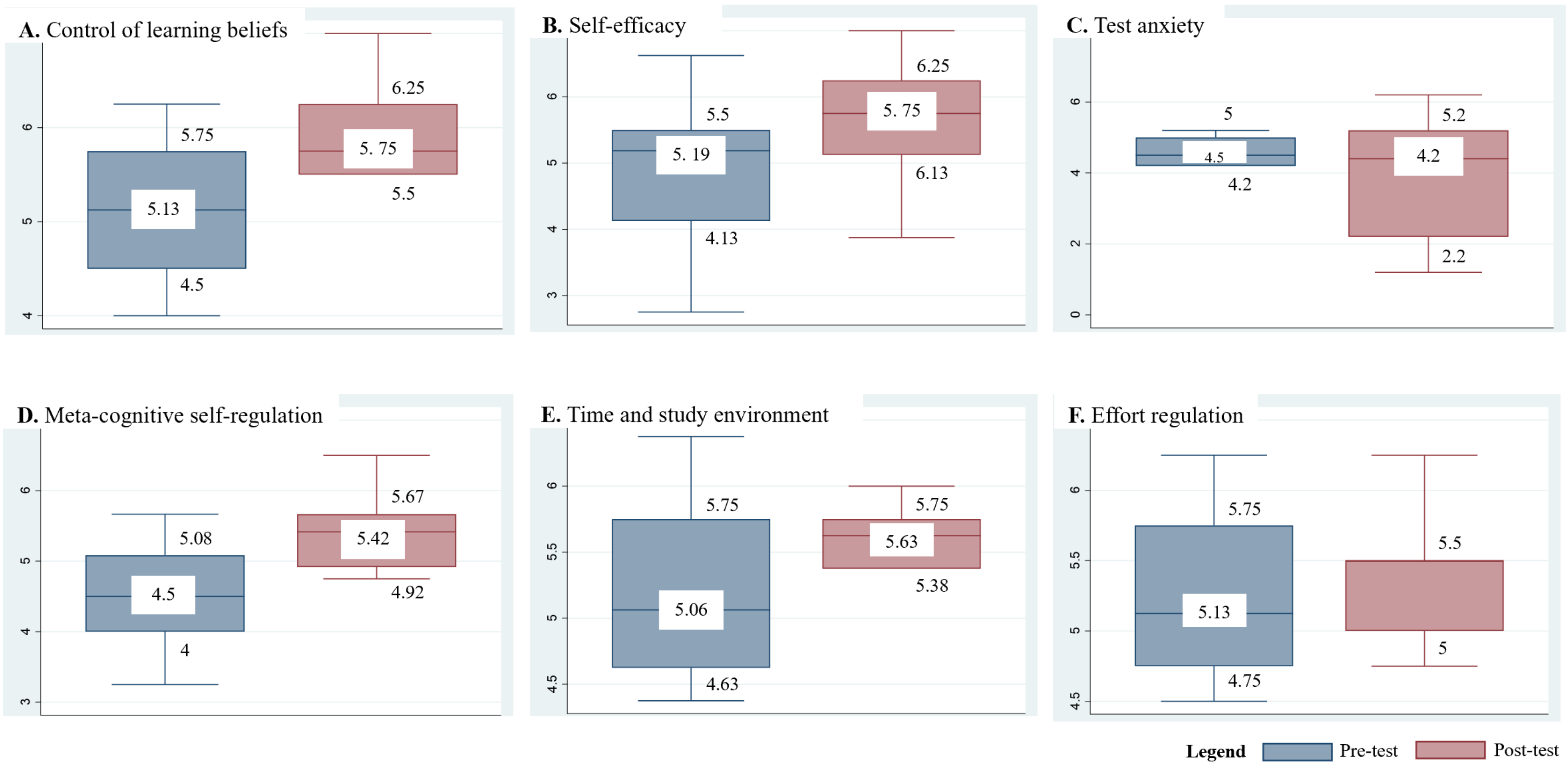

Figure S2. Box plot of motivated strategies for learning subscale scores.

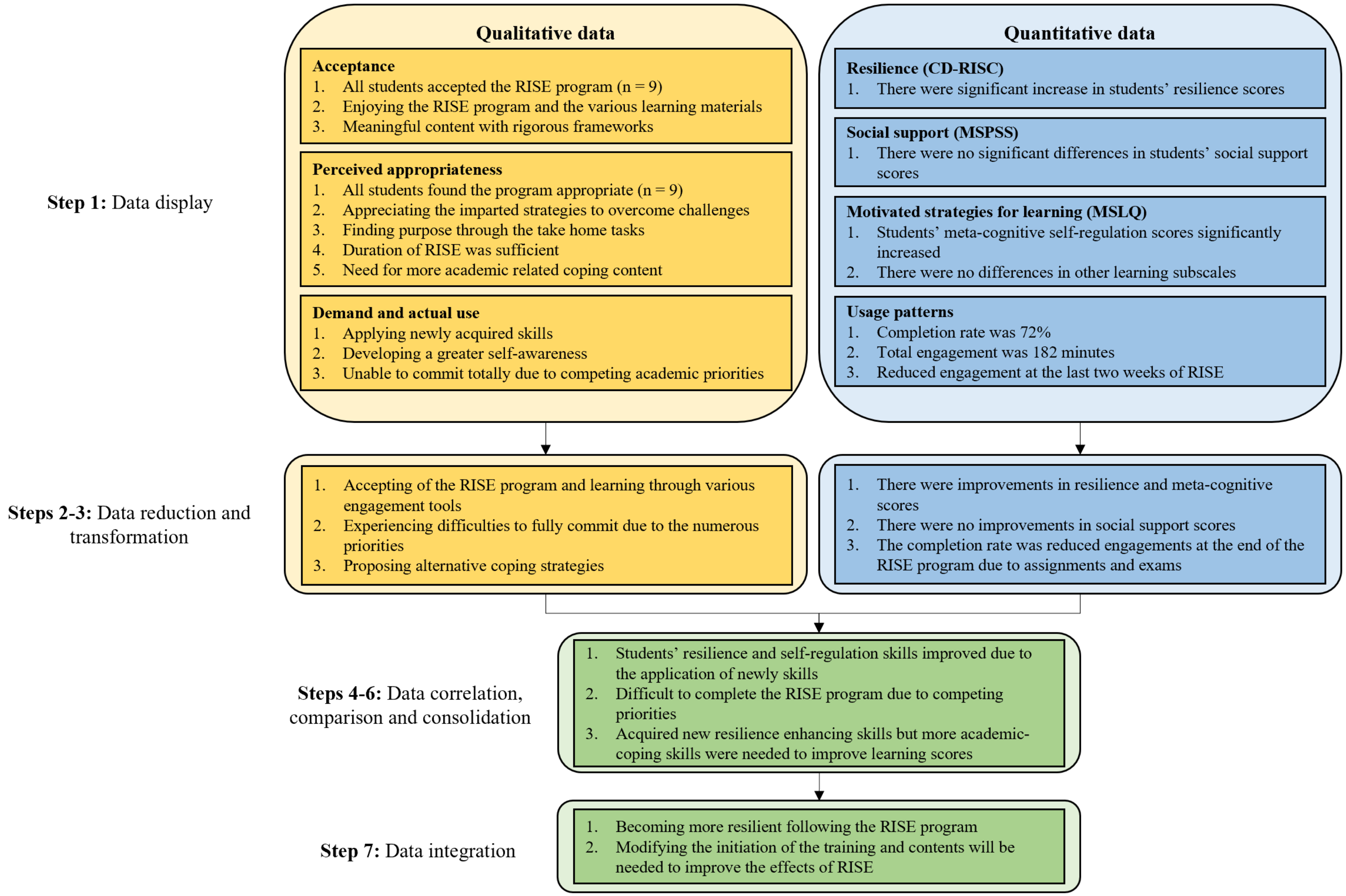

Figure S3. Evaluation of RISE program using a mixed data analysis.
